# Supplementary material for: Deciphering a critical role of uterine epithelial SHP2 in parturition initiation at single cell resolution
Source: Nat Commun. 2023 Nov 14;14:7356. doi: 10.1038/s41467-023-43102-8 (PMC10646072; doi:10.1038/s41467-023-43102-8)
Supplement: Supplementary file 1 — Supplementary Information [file 41467_2023_43102_MOESM1_ESM.pdf]

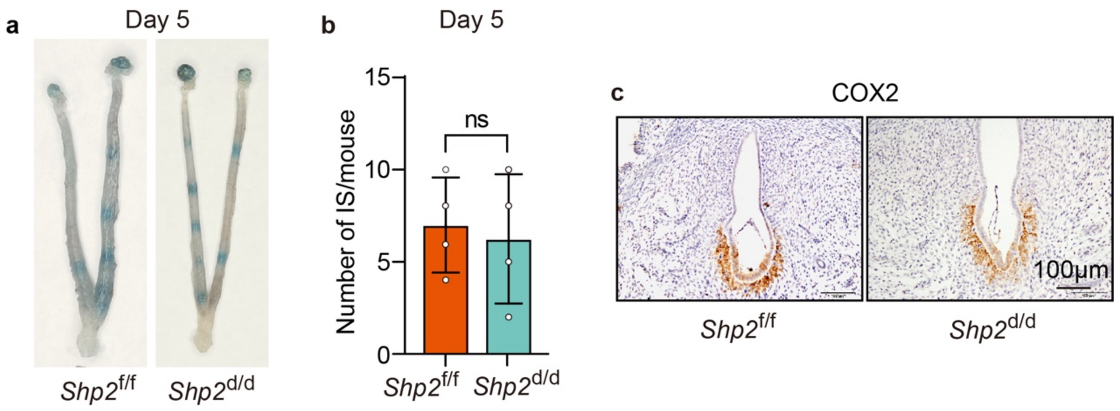


**Supplementary Figure. 1 Mice with epithelial *Shp2* deletion show normal embryo implantation.**

**a**, Implantation marked by Chicago blue dye solution in *Shp2*$^{f/f}$ and *Shp*2$^{d/d}$ mice on day 5.

**b**, The average number of implantation sites in *Shp2*$^{f/f}$ (n=4 animals) and *Shp*2$^{d/d}$ (n=4 animals) mice on day 5. Data represent the mean ± SEM. Two-tailed unpaired student's *t*-test, ns, not significant.

**c**, Immunohistochemical analysis of COX2 in *Shp2*$^{f/f}$ and *Shp*2$^{d/d}$ mice implantation sites on day 5. This result was repeated independently in three individual mice with similar results.

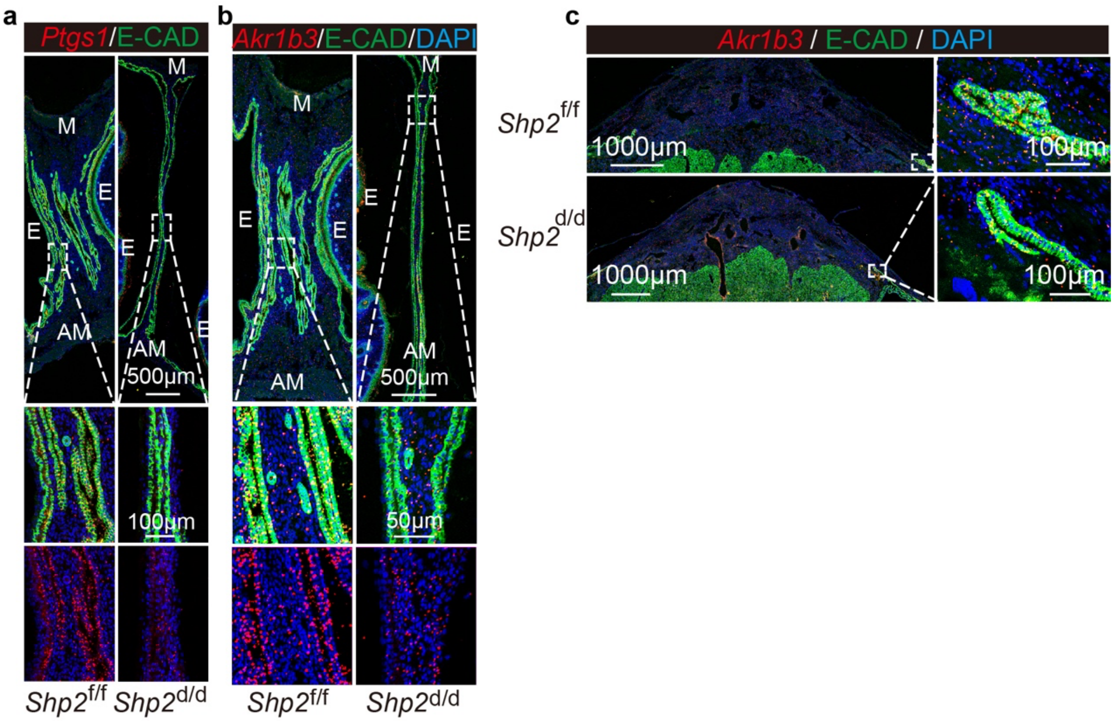


**Supplementary Figure. 2 Epithelial *Shp2*-deficient uteri displayed abnormal production of PGF2α.**

**a**, Sm-FISH of *Ptgs1* in *Shp2*$^{f/f}$ and *Shp*2$^{d/d}$ mice inter-implantation sites on day 19 with immunofluorescence staining of E-cadherin. This result was repeated independently in three individual mice with similar results.

**b**, Sm-FISH of *Akr1b3* in *Shp2*$^{f/f}$ and *Shp*2$^{d/d}$ mice inter-implantation sites on day 19 with immunofluorescence staining of E-cadherin. M: mesometrial; AM: antimesometrial; E: embryos. This result was repeated independently in three individual mice with similar results.

**c**, Sm-FISH of *Akr1b3* in *Shp2*$^{f/f}$ and *Shp*2$^{d/d}$ mice implantation sites on day 19 with immunofluorescence staining of E-cadherin. This result was repeated independently in three individual mice with similar results.

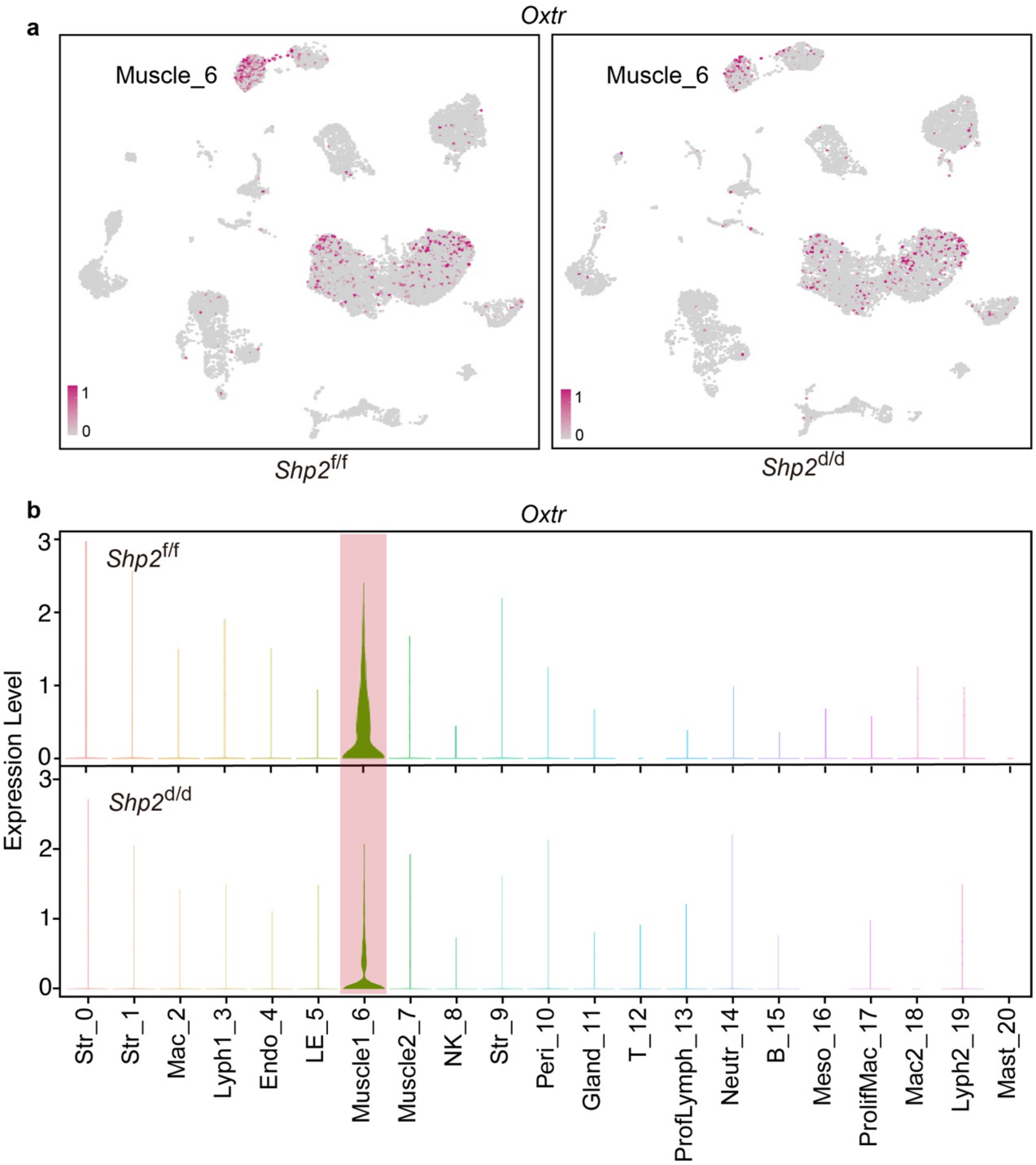


**Supplementary Figure. 3 The expression of *Oxtr* in *Shp2*$^{f/f}$ and *Shp2*$^{d/d}$ mice on day 19.**

**a**, UMAP visualization of the expression of *Oxtr* in day 19 *Shp2*$^{f/f}$ and *Shp2*$^{d/d}$ uteri. Color bar represents expression of indicated gene in cells.

**b**, Violin plot showing expression of *Oxtr* in *Shp2*$^{f/f}$ and *Shp2*$^{d/d}$ mice on day 19.

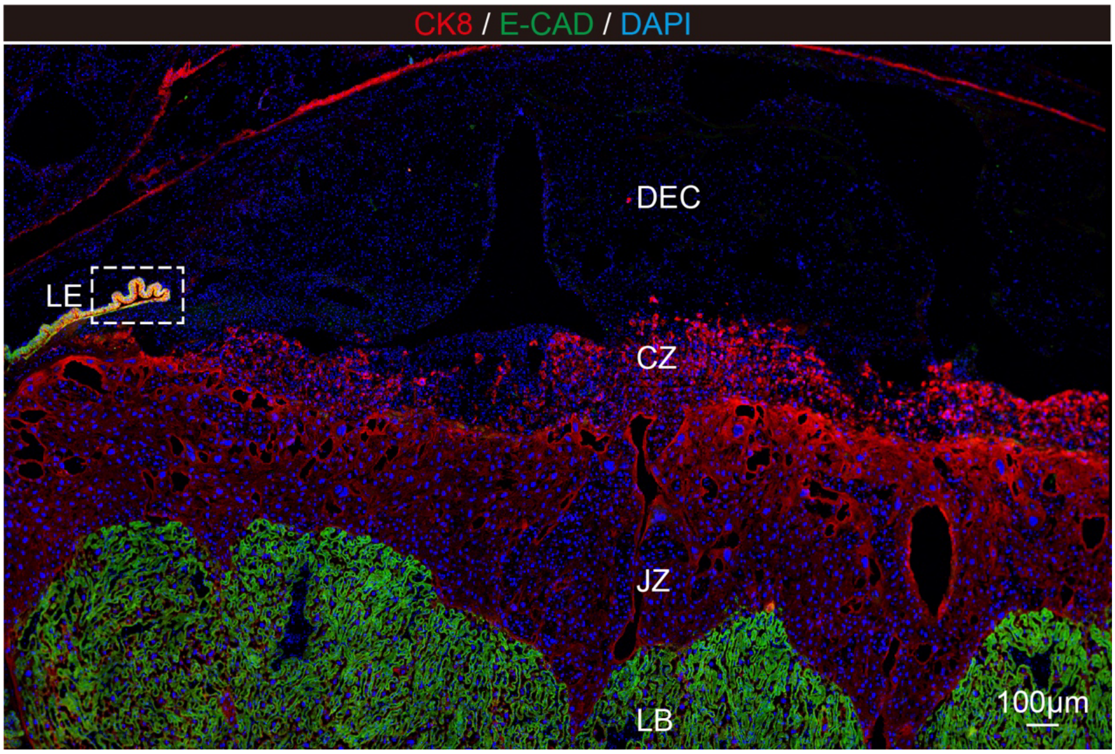


**Supplementary Figure. 4 The expression of CK8 and E-cadherin in day 16 mouse uteri.**

Immunofluorescence staining of CK8 (red) and E-cadherin (green) in day 16 mouse uteri. LE: luminal epithelum; DEC: decidua; CZ: compacting zone; JZ: junctional zone; LB: labyrinth zone.

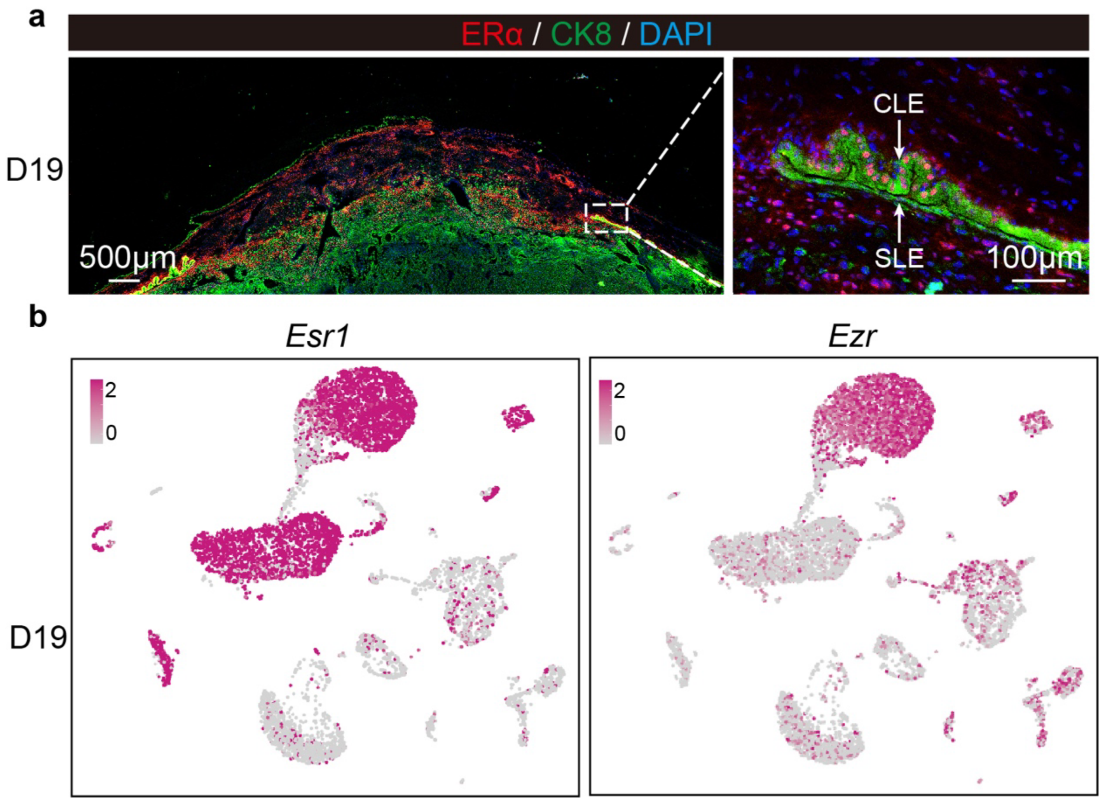


**Supplementary Figure. 5 The expression of *Esr1* and *Ezr* in day 19 mouse uteri.**

**a**, Immunofluorescence staining of ERα and CK8 in day 19 mouse uteri. CLE: columnar luminal epithelium; SLE: squamous luminal epithelium. This result was repeated independently in three individual mice with similar results.

**b**, UMAP visualization of the expression of *Esr1* and *Ezr* in day 19 mouse uteri. Color bar represents expression of indicated gene in cells.

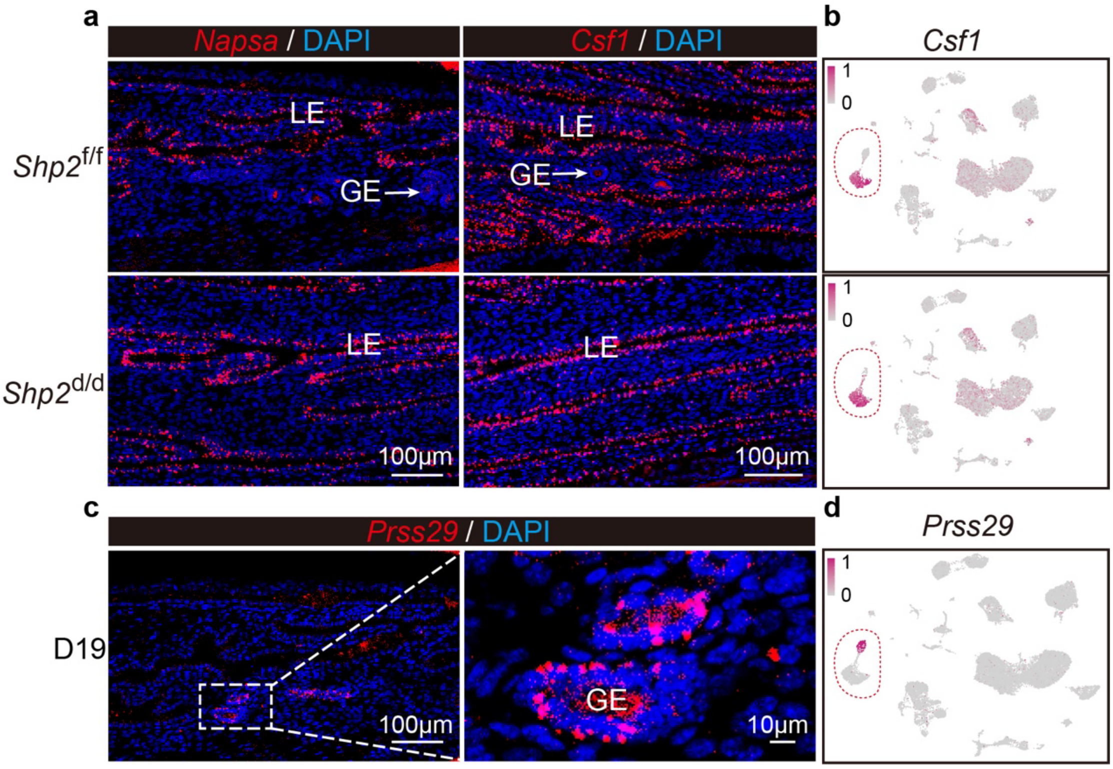


**Supplementary Figure. 6 The marker of luminal epithelium and glandular epithelium in day 19 mouse uteri.**

**a-b**, Sm-FISH and UMAP of *Napsa* and *Csf1* in *Shp*2$^{f/f}$ and *Shp*2$^{d/d}$ mice inter-implantation sites on day 19. LE: luminal epithelium; GE: glandular epithelium. The result of **a** was repeated independently in three individual mice with similar results. Color bar represents expression of indicated gene in cells.

**c-d**, Sm-FISH and UMAP of *Prss29* in mice inter-implantation sites on day 19. GE: glandular epithelium. The result of **c** was repeated independently in three individual mice with similar results. Color bar represents expression of indicated gene in cells.

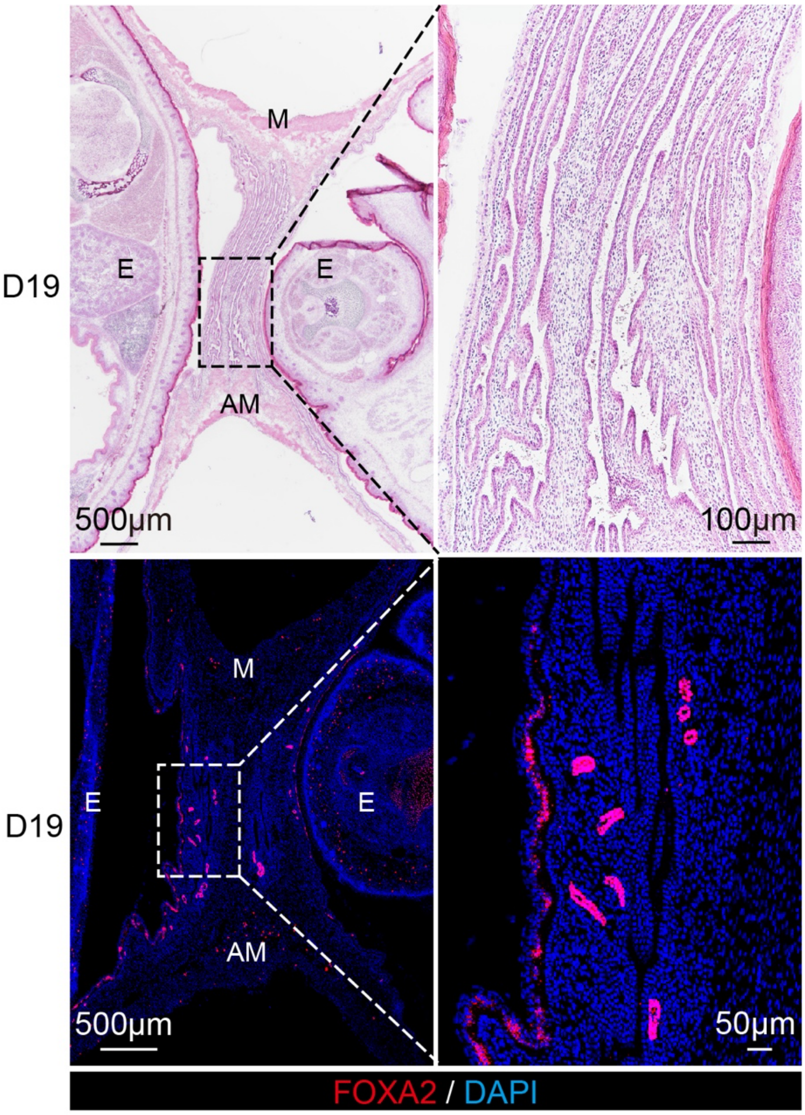


**Supplementary Figure. 7 The location of glands in day 19 mouse uteri.** Histology of glands and immunofluorescence staining of FOXA2 in day 19 mouse uteri. M: mesometrial; AM: antimesometrial; E: embryos. This result was repeated independently in three individual mice with similar results.

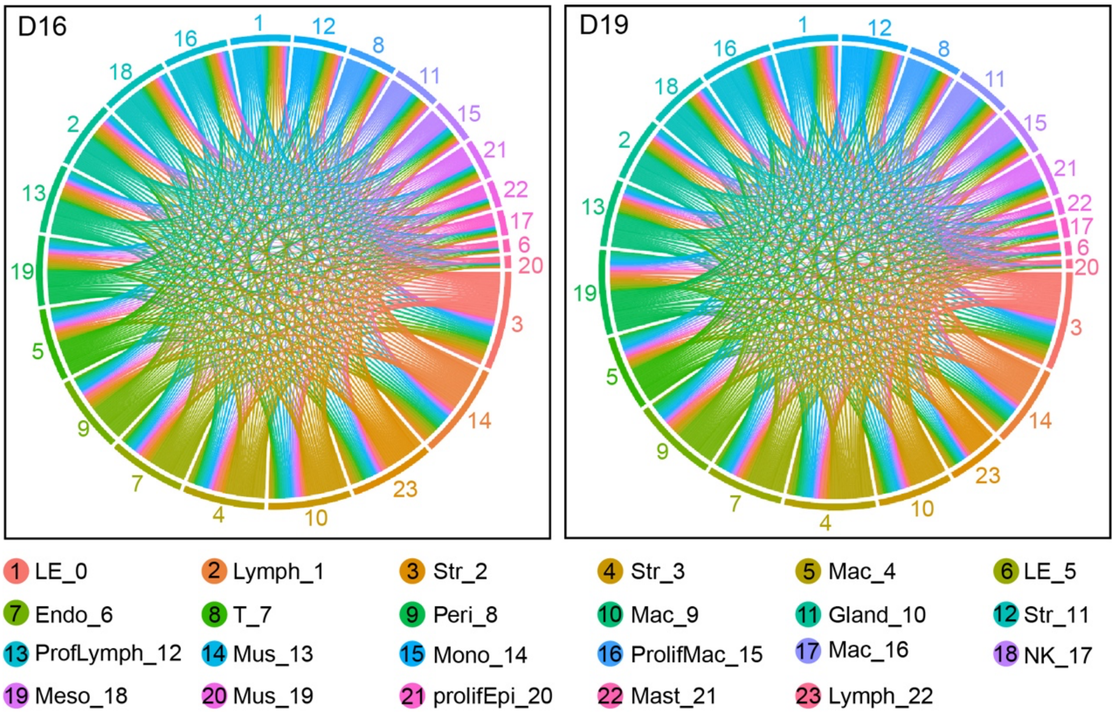


**Supplementary Figure. 8 Cell interactions in days 16 and 19 mouse uteri.** The circular plot showing sending and accepting signaling in all cell types in days 16 and 19 mouse uteri utilizing CellphoneDB.

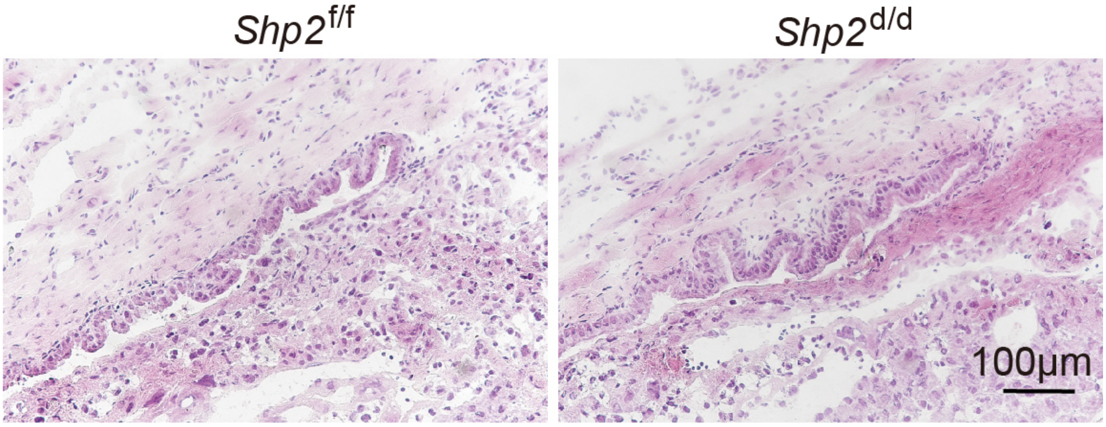


**Supplementary Figure. 9 Epithelial morphology in *Shp2*$^{f/f}$ and *Shp*2$^{d/d}$ mouse uteri.**

Histology of epithelium of *Shp2*$^{f/f}$ and *Shp*2$^{d/d}$ mouse uteri on day 19. This result was repeated independently in three individual mice with similar results.

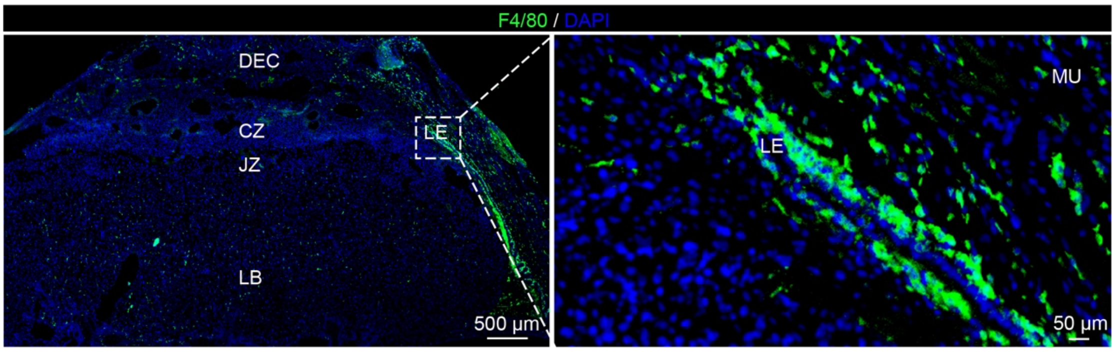


**Supplementary Figure. 10 The location of macrophages in days 16 mouse uteri.**

Immunostaining of F4/80 in day 16 uteri. LE: luminal epithelum; DEC: decidua; CZ: compacting zone; JZ: junctional zone; LB: labyrinth zone, MU: muscle. This result was repeated independently in three individual mice with similar results.

Supplementary Table 1 Antibodies used for immunohistochemistry (IHC), immunofluorescence (IF), western blotting (WB)

| Antigen | Reactivity | Clone | Cat | Manufacturer | Application |
|---|---|---|---|---|---|
| COX2 | mouse | D5H5 | 12282s | Cell Signaling Technology | IHC |
| STAR | mouse | | Ab58013 | Abcam | WB |
| SHP2 | mouse | | sc-280 | Santa Cruz | IF |
| CK8 | mouse | | AB_531826 | DSHB | IF |
| FOXA2 | mouse | EPR4466 | ab108422 | Abcam | IF |
| COX1 | mouse | | ab109025 | Abcam | IF/WB |
| PR | mouse | D8Q2J | 8757S | Cell Signaling Technology | IF |
| F4/80 | mouse | | 30325 | Cell Signaling Technology | IF |
| ERα | mouse | | sc-542 | Santa Cruz Biotechnolog | IF |
| KI67 | mouse | | GB121141 | Servicebio | IF |
| E-cadherin | mouse | 24E10 | 3199S | Cell Signaling Technology | IF |
| β-Actin | mouse | | AC038 | Cell Signaling Technology | WB |

Supplementary Table 2 In situ hybridization probes

| Gene name | Forward Primer (5'-3') | Reverse Primer (5'- 3') |
|---|---|---|
| *Akr1c18* | TAATACGACTCACTATAGGGTCCAATTGGCTTCAGATGAC | AATTAACCCTCACTAAAGGGATTGAATTAACTGGGGGTGA |

Supplementary Table 3 Sm-FISH padlock probes

| Gene name | Probe sequence (5'-3') |
|---|---|
| *Napsa* | CCTCACTAGGTCCTGTGATTCCTCTATGATTACTGACTGCGTCTATTTAGTGGAGCCGCCCCTATCTTCTTTTTTATTCAAGGCCCGGATCT |
| | CTAAAGCAAAGATCAGGCTGTCCTCTATGATTACTGACTGCGTCTATTTAGTGGAGCCGCCCCTATCTTCTTTAGGATCCCATCAAAGTGGG |
| *Akr1b7* | TTTGCCTTTATTGTCTTTGGTCCTCTATGATTACTGACTGCGTCTATTTAGTGGAGCCGCCCCTATCTTCTTTTGGATTTACTCAGGAGAAC |
| | GATAGTCCTCTTCAGTCCTTTCCTCTATGATTACTGACTGCGTCTATTTAGTGGAGCCGCCCCTATCTTCTTTTCAGTATTCCTCGTGGAAAG |
| *Gpx2* | TAGGGCAGCTTGTCTTTCAGTCCTCTATGATTACTGACTGCGTCTATTTAGTGGAGCCGCCCCTATCTTCTTTAGAACGGGTCATCATAAGGG |
| | CCCAGAAGGGTTTAGGAATCCTCTATGATTACTGACTGCGTCTATTTAGTGGAGCCGCCCCTATCTTCTTTTCACAACTTCTCAGACATCT |
| *Ptgs1* | TAGAGATGGTTAAATTCCATGTCCTCTATGATTACTGACTGCGTCTATTTAGTGGAGCCGCCCCTATCTTCTTTATGAGTGGATGCCAGTGA |
| | CAACAAAGAGCCAAGTTCTGTCCTCTATGATTACTGACTGCGTCTATTTAGTGGAGCCGCCCCTATCTTCTTTAAATCTGACTTTCTGAGTTGC |
| | TCATCCGAGAAGTACTCATGTCCTCTATGATTACTGACTGCGTCTATTTAGTGGAGCCGCCCCTATCTTCTTTGGAATTTGTGAATGCCACCT |
| *Fabp4* | TCCACAAGAGTTTATGAAAGTCCTCTATGATTACTGACTGCGTCTATTTAGTGGAGCCGCCCCTATCTTCTTTTGTTATGAAAGGCGTGACT |
| | CTTTCATGTAATCATCGAAGTTCCTCTATGATTACTGACTGCGTCTATTTAGTGGAGCCGCCCCTATCTTCTTTAAAGCCCACTCCCACTT |
| | GTCGACCACAATAAAGAGAAATCCTCTATGATTACTGACTGCGTCTATTTAGTGGAGCCGCCCCTATCTTCTTTTGCAGAAGTGGGATGGAAA |
| *Prl8a2* | CCTCATCACGTCTATACATGTCCTCTATGATTACTGACTGCGTCTATTTAGTGGAGCCGCCCCTATCTTCTTTATTTCTGGCTCTGAGAACCT |
| | GGTATTCCCATATGATAGATTCCTCTATGATTACTGACTGCGTCTATTTAGTGGAGCCGCCCCTATCTTCTTTTTGATCTTAGGCCTGGAA |
| *Akr1b3* | TCTGAAGAGGGTTGAAGTTTCCTCTATGATTACTGACTGCGTCTATTTAGTGGAGCCGCCCCTATCTTCTTTTTTGTTCAAGATCCTCTCAA |
| | ATTGCAGCCAAGTACAATAAATCCTCTATGATTACTGACTGCGTCTAT |

| | |
|---|---|
| | TTAGTGGAGCCGCCCCTATCTTCTTTGATCCCAGGATCAAAGCA |
| | ATCAGCTTCTCCTGAGTTAGTCCTCTATGATTACTGACTGCGTCTATTTAGTGGAGCCGCCCCTATCTTCTTTCTTTGCTGTGGCAGTATTCA |
| *Csf1* | TGATGCCCTCTGTTTCCCATCCTCTATGATTACTGACTGCGTCTATTTAGTGGAGCCGCCCCTATCTTCTTTGTTGTTTACTTCCAACTTGTTC |
| | TCCATAAAGAGATAGTCCTGTCCTCTATGATTACTGACTGCGTCTATTTAGTGGAGCCGCCCCTATCTTCTTTATGTTCCCATATGTCTCCT |
| | TCTATTATGTCTTGTACCAGTCCTCTATGATTACTGACTGCGTCTATTTAGTGGAGCCGCCCCTATCTTCTTTTTTAAAGCGCATGGTCTCA |
| *Prss29* | GTGAGTTGTCAATTATCTTTACTCCTCTATGATTACTGACTGCGTCTATTTAGTGGAGCCGCCCCTATCTTCTTTTGGTACATCTCCTCACAAA |
| | AAAGGATTGTACAGACACTGTCCTCTATGATTACTGACTGCGTCTATTTAGTGGAGCCGCCCCTATCTTCTTTTGACTGGCTTGACATTAGG |
| | TCTCTCTCACGAATGCAGTGTCCTCTATGATTACTGACTGCGTCTATTTAGTGGAGCCGCCCCTATCTTCTTTAAAGACTGATGGGTCGGCA |
| *Spp1* | AAGATGAACTCTCTAATTCATGTCCTCTATGATTACTGACTGCGTCTATTTAGTGGAGCCGCCCCTATCTTCTTTTCTTCTTTAGTTGACCTCAG |
| | AAGTGTCTGCTTGTGTACTAGTCCTCTATGATTACTGACTGCGTCTATTTAGTGGAGCCGCCCCTATCTTCTTTGTAGGGACGATTGGAGTGA |
| | CTCATTTCTCAGTTCAGTGTCCTCTATGATTACTGACTGCGTCTATTTAGTGGAGCCGCCCCTATCTTCTTTTTAAGCAGGAATACTAACTG |

Supplementary Table 4 Organoid medium compositions

| reagent | Cat | Final concentration |
|---|---|---|
| DMEM/F12 | Gibco 11039-021 | |
| penicillin/streptomycin | Gibco 15140-122 | 1% |
| ITS | Gibco 41400-045 | 1% |
| L-Glutamine | Gibco 21051-024 | 2 mM |
| Nicotinamide | Sigma N3376 | 1 mM |
| B27 | Gibco 17504-044 | 2% |
| N2 | Gibco 17502-048 | 1% |
| EGF | Peprotech 315-09 | 50 ng/ml |
| FGF-10 | Peprotech 100-26 | 100 ng/ml |
| Noggin | Peprotech 250-38 | 100 ng/ml |
| WNT-3A | Peprotech 315-20 | 200 ng/ml |
| R-Spondin-1 | Peprotech 120-38 | 200 ng/ml |
| A83-01 | MCE HY-10432 | 0.5 μM |
| Nac | Sigma A7250 | 1.25 mM |
| Y27632 | WAKO | 10 μM |
