# Supplementary material for: Deciphering a critical role of uterine epithelial SHP2 in parturition initiation at single cell resolution
Source: Nat Commun. 2023 Nov 14;14:7356. doi: 10.1038/s41467-023-43102-8 (PMC10646072; doi:10.1038/s41467-023-43102-8)
Supplement: Supplementary file 3 — Reporting Summary [file 41467_2023_43102_MOESM3_ESM.pdf]

nature portfolio

Corresponding author(s): Wenbo Deng

Last updated by author(s): Oct 19, 2023

Reporting Summary

Nature Portfolio wishes to improve the reproducibility of the work that we publish. This form provides structure for consistency and transparency in reporting. For further information on Nature Portfolio policies, see our Editorial Policies and the Editorial Policy Checklist.

Statistics

For all statistical analyses, confirm that the following items are present in the figure legend, table legend, main text, or Methods section.

n/a	Confirmed	
☐	☒	The exact sample size (*n*) for each experimental group/condition, given as a discrete number and unit of measurement
☐	☒	A statement on whether measurements were taken from distinct samples or whether the same sample was measured repeatedly
☐	☒	The statistical test(s) used AND whether they are one- or two-sided<br>*Only common tests should be described solely by name; describe more complex techniques in the Methods section.*
☐	☒	A description of all covariates tested
☐	☒	A description of any assumptions or corrections, such as tests of normality and adjustment for multiple comparisons
☐	☒	A full description of the statistical parameters including central tendency (e.g. means) or other basic estimates (e.g. regression coefficient) AND variation (e.g. standard deviation) or associated estimates of uncertainty (e.g. confidence intervals)
☐	☒	For null hypothesis testing, the test statistic (e.g. *F*, *t*, *r*) with confidence intervals, effect sizes, degrees of freedom and *P* value noted<br>*Give P values as exact values whenever suitable.*
☒	☐	For Bayesian analysis, information on the choice of priors and Markov chain Monte Carlo settings
☒	☐	For hierarchical and complex designs, identification of the appropriate level for tests and full reporting of outcomes
☒	☐	Estimates of effect sizes (e.g. Cohen's *d*, Pearson's *r*), indicating how they were calculated

Our web collection on statistics for biologists contains articles on many of the points above.

Software and code

Policy information about availability of computer code

Data collection	Leica DM2500 light microscope and Zeiss LSM 880+Airyscan for image capturing<br>ChemiDocTMXRS+ (BIO-RAD) for western blot
Data analysis	Bowtie2(2.5.0)<br>MACS2(2.2.7.1)<br>Seurat (4.3.0)<br>CellphoneDB(v4)<br>Cellchat(1.5.0)<br>NicheNet (1.1.1)<br>TopHat2 (2.1.1)<br>GraphPad Prism (8.0.0)

For manuscripts utilizing custom algorithms or software that are central to the research but not yet described in published literature, software must be made available to editors and reviewers. We strongly encourage code deposition in a community repository (e.g. GitHub). See the Nature Portfolio guidelines for submitting code & software for further information.

Data

Policy information about availability of data

All manuscripts must include a data availability statement. This statement should provide the following information, where applicable:

- Accession codes, unique identifiers, or web links for publicly available datasets
- A description of any restrictions on data availability
- For clinical datasets or third party data, please ensure that the statement adheres to our policy

Data availability
Raw data generated in this study have been deposited at the National Center for Biotechnology Information Sequence Read Archive under accession number PRJNA936400 (https://www.ncbi.nlm.nih.gov/sra?term=SRP423274). Source data are provided with this paper.

Code availability
Codes used in this study have been deposited at Zenodo (DOI: 10.5281/zenodo.10022179).

Research involving human participants, their data, or biological material

Policy information about studies with human participants or human data. See also policy information about sex, gender (identity/presentation), and sexual orientation and race, ethnicity and racism.

Reporting on sex and gender	N.A.
Reporting on race, ethnicity, or other socially relevant groupings	N.A.
Population characteristics	N.A.
Recruitment	N.A.
Ethics oversight	N.A.

Note that full information on the approval of the study protocol must also be provided in the manuscript.

Field-specific reporting

Please select the one below that is the best fit for your research. If you are not sure, read the appropriate sections before making your selection.

☒ Life sciences ☐ Behavioural & social sciences ☐ Ecological, evolutionary & environmental sciences

For a reference copy of the document with all sections, see nature.com/documents/nr-reporting-summary-flat.pdf

Life sciences study design

All studies must disclose on these points even when the disclosure is negative.

Sample size	For animal work, at least 3 independent mice were used for each experimental group. The sample sizes of high-throughput sequencing were based on previously published openly shared datasets found in various sources. For in vitro experiments, sample sizes were determined based on our pilot experiments.
Data exclusions	No data were excluded.
Replication	All in vivo and in vitro functional and phenotypic experiments were performed in at least biological triplicate to ensure reproducibility. Similar results were obtained in at least three independent experiments.
Randomization	All samples were allocated at random.
Blinding	We were blinded to group allocation during data collection.

Reporting for specific materials, systems and methods

We require information from authors about some types of materials, experimental systems and methods used in many studies. Here, indicate whether each material, system or method listed is relevant to your study. If you are not sure if a list item applies to your research, read the appropriate section before selecting a response.

Materials & experimental systems

n/a	Involved in the study
☐	☒ Antibodies
☐	☐ Eukaryotic cell lines
☐	☐ Palaeontology and archaeology
☐	☒ Animals and other organisms
☐	☐ Clinical data
☐	☐ Dual use research of concern
☐	☐ Plants

Methods

n/a	Involved in the study
☐	☐ ChIP-seq
☐	☐ Flow cytometry
☐	☐ MRI-based neuroimaging

Antibodies

Antibodies used

For immunostaining, following antibodies were used
SHP2(sc-280, Santa Cruz,1:200)
CK8(AB_531826,DSHB,1:500)
FOXA2(ab108422, Abcam,EPR4466,1:500)
PR(8757S, Cell Signaling Technology,D8Q2J,1:200)
F4/80(30325, Cell Signaling Technology,1:200)
ERα(sc-542, Santa Cruz Biotechnolog,1:500)
KI67(GB121141, Servicebio,1:200)
E-cadherin(3199S, Cell Signaling Technology,24E10,1:200)
COX2(12282s,Cell Signaling Technology,D5H5,1:200)
Anti-Digoxigenin-AP, Fab fragments(1093274910, Roche,1:2500)
Cy™3 AffiniPure Goat Anti-Rabbit IgG (H+L) (711-165-152, Jackson ImmunoResearch,1:200)
Cy™2 AffiniPure Goat Anti-Rabbit IgG (H+L) (711-225-152, Jackson ImmunoResearch,1:200)
Cy™2 AffiniPure Goat Anti-Rat IgG (H+L) (712-225-153, Jackson ImmunoResearch,1:200)

For Western blotting, the following antibodies were used:
β-Actin (AC038,Cell Signaling Technology,1:3000)
STAR(Ab58013,Abcam,1:1000)
COX1(ab109025, Abcam,1:1000)

Validation

All the other antibodies used have been extensively utilized in the literature and have been validated previously. All antibodies were commercially available. Listed below are information available.
SHP2(sc-280, Santa Cruz): https://www.scbt.com/p/sh-ptp2-antibody-c-18
CK8(AB_531826,DSHB,https://dshb.biology.uiowa.edu/TROMA-I)
COX1(ab109025, Abcam):https://www.abcam.com/products/primary-antibodies/cox1--cyclooxygenase-1-antibody-epr5866-ab109025.html
F4/80(30325, Cell Signaling Technology):https://www.cellsignal.com/products/primary-antibodies/f4-80-d4c8v-xp-rabbit-mab/30325
ERα(sc-542, Santa Cruz Biotechnolog):https://www.scbt.com/p/eralpha-antibody-mc-20
E-cadherin(3199S, Cell Signaling Technology):https://www.cellsignal.com/products/antibody-conjugates/e-cadherin-24e10-rabbit-mab-alexa-fluor-488-conjugate/3199
FOXA2(ab108422, Abcam):https://www.abcam.com/products/primary-antibodies/foxa2-antibody-epr4466-ab108422.html
COX2 (sc-1746, Santa Cruz): https://www.scbt.com/p/cox-2-antibody-n-20
KI-67 (GB121141, Servicebio): https://www.servicebio.cn/goodsdetail?id=6801
PR (8757, Cell Signaling Technology): https://www.cellsignal.cn/products/primary-antibodies/progesterone-receptor-a-b-d8q2j-xp-rabbit-mab/8757
β-Actin (AP0060, Bioworld): https://www.bioworlde.com/Primary-Antibodies/25097.html
Anti-Digoxigenin-AP, Fab fragments (1093274910, Roche) https://www.sigmaaldrich.cn/CN/zh/product/roche/11093274910
Cy™3 AffiniPure Goat Anti-Rabbit IgG (H+L) (711-165-152, Jackson ImmunoResearch)https://www.jacksonimmuno.com/catalog/products/711-165-152
Cy™2 AffiniPure Goat Anti-Rabbit IgG (H+L) (711-225-152, Jackson ImmunoResearch)https://www.jacksonimmuno.com/catalog/products/711-225-152
Cy™2 AffiniPure Goat Anti-Rat IgG (H+L) (711-225-152, Jackson ImmunoResearch)https://www.jacksonimmuno.com/catalog/products/712-225-153

Eukaryotic cell lines

Policy information about cell lines and Sex and Gender in Research

Cell line source(s)

State the source of each cell line used and the sex of all primary cell lines and cells derived from human participants or vertebrate models.

Authentication

Describe the authentication procedures for each cell line used OR declare that none of the cell lines used were authenticated.

Mycoplasma contamination

Confirm that all cell lines tested negative for mycoplasma contamination OR describe the results of the testing for mycoplasma contamination OR declare that the cell lines were not tested for mycoplasma contamination.

Commonly misidentified lines (See ICLAC register)	*Name any commonly misidentified cell lines used in the study and provide a rationale for their use.*

Palaeontology and Archaeology

Specimen provenance	*Provide provenance information for specimens and describe permits that were obtained for the work (including the name of the issuing authority, the date of issue, and any identifying information). Permits should encompass collection and, where applicable, export.*
Specimen deposition	*Indicate where the specimens have been deposited to permit free access by other researchers.*
Dating methods	*If new dates are provided, describe how they were obtained (e.g. collection, storage, sample pretreatment and measurement), where they were obtained (i.e. lab name), the calibration program and the protocol for quality assurance OR state that no new dates are provided.*

☐ Tick this box to confirm that the raw and calibrated dates are available in the paper or in Supplementary Information.

Ethics oversight	*Identify the organization(s) that approved or provided guidance on the study protocol, OR state that no ethical approval or guidance was required and explain why not.*

Note that full information on the approval of the study protocol must also be provided in the manuscript.

Animals and other research organisms

Policy information about studies involving animals; ARRIVE guidelines recommended for reporting animal research, and Sex and Gender in Research

Laboratory animals	2-month-old LtfiCre/+;Shp2flox/flox female mice
Wild animals	N.A.
Reporting on sex	Only females were used in this study for parturition study.
Field-collected samples	2-month-old LtfiCre/+;Shp2flox/flox and Shp2flox/flox female mice in C57B6 background were used in the present study. Mice were housed in the animal care facility of Xiamen University with controlled environment (22±2°C, 50 -60% humidity, 12-h light/dark cycle, lights on at 7AM) and free access to food and water according to the guidelines for the care and use of laboratory animals.
Ethics oversight	Mice were housed in the animal care facility of Xiamen University according to the guidelines for the care and use of laboratory animals. All experimental procedures were approved by the Animal Welfare Committee of Research Organization (X200811), Xiamen University.

Note that full information on the approval of the study protocol must also be provided in the manuscript.

Clinical data

Policy information about clinical studies
All manuscripts should comply with the ICMJE guidelines for publication of clinical research and a completed CONSORT checklist must be included with all submissions.

Clinical trial registration	*Provide the trial registration number from ClinicalTrials.gov or an equivalent agency.*
Study protocol	*Note where the full trial protocol can be accessed OR if not available, explain why.*
Data collection	*Describe the settings and locales of data collection, noting the time periods of recruitment and data collection.*
Outcomes	*Describe how you pre-defined primary and secondary outcome measures and how you assessed these measures.*

Dual use research of concern

Policy information about dual use research of concern

Hazards

Could the accidental, deliberate or reckless misuse of agents or technologies generated in the work, or the application of information presented in the manuscript, pose a threat to:

No	Yes	
☒	☐	Public health
☒	☐	National security
☒	☐	Crops and/or livestock
☒	☐	Ecosystems
☒	☐	Any other significant area

Experiments of concern

Does the work involve any of these experiments of concern:

No	Yes	
☒	☐	Demonstrate how to render a vaccine ineffective
☒	☐	Confer resistance to therapeutically useful antibiotics or antiviral agents
☒	☐	Enhance the virulence of a pathogen or render a nonpathogen virulent
☒	☐	Increase transmissibility of a pathogen
☒	☐	Alter the host range of a pathogen
☒	☐	Enable evasion of diagnostic/detection modalities
☒	☐	Enable the weaponization of a biological agent or toxin
☒	☐	Any other potentially harmful combination of experiments and agents

Plants

Seed stocks	*Report on the source of all seed stocks or other plant material used. If applicable, state the seed stock centre and catalogue number. If plant specimens were collected from the field, describe the collection location, date and sampling procedures.*
Novel plant genotypes	*Describe the methods by which all novel plant genotypes were produced. This includes those generated by transgenic approaches, gene editing, chemical/radiation-based mutagenesis and hybridization. For transgenic lines, describe the transformation method, the number of independent lines analyzed and the generation upon which experiments were performed. For gene-edited lines, describe the editor used, the endogenous sequence targeted for editing, the targeting guide RNA sequence (if applicable) and how the editor was applied.*
Authentication	*Describe any authentication procedures for each seed stock used or novel genotype generated. Describe any experiments used to assess the effect of a mutation and, where applicable, how potential secondary effects (e.g. second site T-DNA insertions, mosiacism, off-target gene editing) were examined.*

ChIP-seq

Data deposition

☐ Confirm that both raw and final processed data have been deposited in a public database such as GEO.

☐ Confirm that you have deposited or provided access to graph files (e.g. BED files) for the called peaks.

Data access links<br>*May remain private before publication.*	*For "Initial submission" or "Revised version" documents, provide reviewer access links. For your "Final submission" document, provide a link to the deposited data.*
Files in database submission	*Provide a list of all files available in the database submission.*
Genome browser session<br>(e.g. UCSC)	*Provide a link to an anonymized genome browser session for "Initial submission" and "Revised version" documents only, to enable peer review. Write "no longer applicable" for "Final submission" documents.*

Methodology

Replicates	*Describe the experimental replicates, specifying number, type and replicate agreement.*
Sequencing depth	*Describe the sequencing depth for each experiment, providing the total number of reads, uniquely mapped reads, length of reads and whether they were paired- or single-end.*
Antibodies	*Describe the antibodies used for the ChIP-seq experiments; as applicable, provide supplier name, catalog number, clone name, and lot number.*
Peak calling parameters	*Specify the command line program and parameters used for read mapping and peak calling, including the ChIP, control and index files used.*

Data quality | *Describe the methods used to ensure data quality in full detail, including how many peaks are at FDR 5% and above 5-fold enrichment.*

Software | *Describe the software used to collect and analyze the ChIP-seq data. For custom code that has been deposited into a community repository, provide accession details.*

Flow Cytometry

Plots

Confirm that:

- [ ] The axis labels state the marker and fluorochrome used (e.g. CD4-FITC).
- [ ] The axis scales are clearly visible. Include numbers along axes only for bottom left plot of group (a 'group' is an analysis of identical markers).
- [ ] All plots are contour plots with outliers or pseudocolor plots.
- [ ] A numerical value for number of cells or percentage (with statistics) is provided.

Methodology

Sample preparation | *Describe the sample preparation, detailing the biological source of the cells and any tissue processing steps used.*

Instrument | *Identify the instrument used for data collection, specifying make and model number.*

Software | *Describe the software used to collect and analyze the flow cytometry data. For custom code that has been deposited into a community repository, provide accession details.*

Cell population abundance | *Describe the abundance of the relevant cell populations within post-sort fractions, providing details on the purity of the samples and how it was determined.*

Gating strategy | *Describe the gating strategy used for all relevant experiments, specifying the preliminary FSC/SSC gates of the starting cell population, indicating where boundaries between "positive" and "negative" staining cell populations are defined.*

- [ ] Tick this box to confirm that a figure exemplifying the gating strategy is provided in the Supplementary Information.

Magnetic resonance imaging

Experimental design

Design type | *Indicate task or resting state; event-related or block design.*

Design specifications | *Specify the number of blocks, trials or experimental units per session and/or subject, and specify the length of each trial or block (if trials are blocked) and interval between trials.*

Behavioral performance measures | *State number and/or type of variables recorded (e.g. correct button press, response time) and what statistics were used to establish that the subjects were performing the task as expected (e.g. mean, range, and/or standard deviation across subjects).*

Acquisition

Imaging type(s) | *Specify: functional, structural, diffusion, perfusion.*

Field strength | *Specify in Tesla*

Sequence & imaging parameters | *Specify the pulse sequence type (gradient echo, spin echo, etc.), imaging type (EPI, spiral, etc.), field of view, matrix size, slice thickness, orientation and TE/TR/flip angle.*

Area of acquisition | *State whether a whole brain scan was used OR define the area of acquisition, describing how the region was determined.*

Diffusion MRI ☐ Used ☐ Not used

Preprocessing

Preprocessing software | *Provide detail on software version and revision number and on specific parameters (model/functions, brain extraction, segmentation, smoothing kernel size, etc.).*

Normalization | *If data were normalized/standardized, describe the approach(es): specify linear or non-linear and define image types used for transformation OR indicate that data were not normalized and explain rationale for lack of normalization.*

Normalization template | *Describe the template used for normalization/transformation, specifying subject space or group standardized space (e.g. original Talairach, MNI305, ICBM152) OR indicate that the data were not normalized.*

Noise and artifact removal | *Describe your procedure(s) for artifact and structured noise removal, specifying motion parameters, tissue signals and physiological signals (heart rate, respiration).*

Volume censoring | *Define your software and/or method and criteria for volume censoring, and state the extent of such censoring.*

Statistical modeling & inference

Model type and settings | *Specify type (mass univariate, multivariate, RSA, predictive, etc.) and describe essential details of the model at the first and second levels (e.g. fixed, random or mixed effects; drift or auto-correlation).*

Effect(s) tested | *Define precise effect in terms of the task or stimulus conditions instead of psychological concepts and indicate whether ANOVA or factorial designs were used.*

Specify type of analysis: ☐ Whole brain ☐ ROI-based ☐ Both

Statistic type for inference | *Specify voxel-wise or cluster-wise and report all relevant parameters for cluster-wise methods.*

(See Eklund et al. 2016)

Correction | *Describe the type of correction and how it is obtained for multiple comparisons (e.g. FWE, FDR, permutation or Monte Carlo).*

Models & analysis

n/a	Involved in the study
☐	☐ Functional and/or effective connectivity
☐	☐ Graph analysis
☐	☐ Multivariate modeling or predictive analysis

Functional and/or effective connectivity | *Report the measures of dependence used and the model details (e.g. Pearson correlation, partial correlation, mutual information).*

Graph analysis | *Report the dependent variable and connectivity measure, specifying weighted graph or binarized graph, subject- or group-level, and the global and/or node summaries used (e.g. clustering coefficient, efficiency, etc.).*

Multivariate modeling and predictive analysis | *Specify independent variables, features extraction and dimension reduction, model, training and evaluation metrics.*
